# Virulence Potential and Genome-Wide Characterization of Drug Resistant *Streptococcus pneumoniae* Clones Selected In Vivo by the 7-Valent Pneumococcal Conjugate Vaccine

**DOI:** 10.1371/journal.pone.0074867

**Published:** 2013-09-19

**Authors:** Nelson Frazão, N. Luisa Hiller, Evan Powell, Josh Earl, Azad Ahmed, Raquel Sá-Leão, Hermínia de Lencastre, Garth D. Ehrlich, Alexander Tomasz

**Affiliations:** 1 Laboratory of Molecular Genetics, Instituto de Tecnologia Química e Biológica, Universidade Nova de Lisboa, Oeiras, Portugal; 2 Allegheny General Hospital, Allegheny-Singer Research Institute, Center for Genomic Sciences, Pittsburgh, Pennsylvania, United States of America; 3 Department of Biological Sciences, Carnegie Mellon University, Pittsburgh, Pennsylvania, United States of America; 4 Laboratory of Molecular Microbiology of Human Pathogens, Instituto de Tecnologia Química e Biológica, Universidade Nova de Lisboa, Oeiras, Portugal; 5 Laboratory of Microbiology, The Rockefeller University, New York, New York, United States of America; Instituto Butantan, Brazil

## Abstract

We used mouse models of pneumococcal colonization and disease combined with full genome sequencing to characterize three major drug resistant clones of *S. pneumoniae* that were recovered from the nasopharynx of PCV7-immunized children in Portugal. The three clones – serotype 6A (ST2191), serotype 15A (ST63) and serotype 19A (ST276) carried some of the same drug resistance determinants already identified in nasopharyngeal isolates from the pre-PCV7 era. The three clones were able to colonize efficiently the mouse nasopharyngeal mucosa where populations of these pneumococci were retained for as long as 21 days. During this period, the three clones were able to asymptomatically invade the olfactory bulbs, brain, lungs and the middle ear mucosa and established populations in these tissues. The virulence potential of the three clones was poor even at high inoculum (10^5^ CFU per mouse) concentrations in the mouse septicemia model and was undetectable in the pneumonia model. Capsular type 3 transformants of clones 6A and 19A prepared in the laboratory produced lethal infection at low cell concentration (10^3^ CFU per mouse) but the same transformants became impaired in their potential to colonize, indicating the importance of the capsular polysaccharide in both disease and colonization. The three clones were compared to the genomes of 56 *S. pneumoniae* strains for which sequence information was available in the public databank. Clone 15A (ST63) only differed from the serotype 19F clone G54 in a very few genes including serotype so that this clone may be considered the product of a capsular switch. While no strain with comparable degree of similarity to clone 19A (ST276) was found among the sequenced isolates, by MLST this clone is a single locust variant (SLV) of Denmark14-ST230 international clone. Clone 6A (ST2191) was most similar to the penicillin resistant Hungarian serotype 19A clone.

## Introduction

The nasopharynx of preschool age children, particularly those attending day-care, is the main ecological niche for the gram-positive pathogen *Streptococcus pneumoniae* that asymptomatically colonizes these individuals at high rates [1]. Colonization is the first step for pneumococcal disease, which may range from non-invasive mucosal infection to severe invasive disease such as sepsis, bacteraemic pneumonia or meningitis [2]. Global estimates indicate that *S. pneumoniae* is responsible for 2 million deaths annually [3].

The development of pneumococcal resistance to penicillin and multiple other drugs is a global concern [4] as it can lead to disease treatment failures [5]. Surprisingly, although *S. pneumoniae* is a genetically diverse species capable of expressing over 90 different capsular types [6], only a limited number of these serotypes associated to a few pandemic clones have been responsible for the increase of drug resistant strains worldwide [4]. The “birthplace” of these drug resistant clones is believed to be the nasopharynx of young children, with a predisposition for upper-respiratory diseases and behavior traits that favor person-to-person contacts. These circumstances, combined with frequent antibiotic use, constitute ideal conditions for the selection, amplification and transmission of drug resistant clones [1].

The first anti-pneumococcal conjugate vaccine was introduced in the United States in 2000, when almost half of all invasive pneumococcal disease (IPD) was caused by pneumococci resistant to penicillin and/or macrolides [7]. The 7-valent pneumococcal conjugate vaccine (PCV7) targeted the most common serotypes causing IPD in children in the US, namely, serotypes 14, 6B, 19F, 18C, 23F, 4, and 9V [8], which also included the serotypes of highly successful drug resistant pandemic clones such as Spain^23F^-1, Spain^6B^-2 or Spain^9V^-3. In virtually all settings, and even with short vaccination schedules, the introduction of PCV7 has led to a significant decline in the frequency of PCV7 serotypes that were often involved with disease before the introduction of this vaccine [9-11].

A secondary but equally important expected outcome of immunization with this conjugate vaccine was to lower drug resistance levels by reducing the frequency of the PCV7 serotypes that were most often associated with antibiotic resistance prior to the introduction of the vaccine [12]. However, immunization with PCV7 has not only led to a serotype replacement but also to the concomitant expansion of drug resistant clones expressing non-PCV7 capsules [13]. The vaccine selected these clones by prevention of *de novo* acquisition of PCV7 serotypes and, at least in some cases, by unmasking of non-PCV7 serotypes [9] which – presumably – were already present in the nasopharynx as minority populations of pneumococci co-colonizing the chidren´s nasopharynx during the pre-vaccine era [9]. These non-PCV7 clones shared the same ecological niche with clones targeted by the vaccine and were consequently exposed to the same environmental insults, namely antibiotic pressure, which may explain their drug resistance patterns.

In 2001-2003, the impact of the PCV7 on colonization and antimicrobial resistance was first investigated in Portugal by sampling the nasopharynx of healthy day-care center attendees. This study documented replacement of PCV7 by non-PCV7 isolates which also carried resistance traits to several antimicrobial agents [14]. Among the drug resistant non-PCV7 pneumococci the dominant serotypes were 6A, 15A and 19A, which – in association with a few clonal lineages – accounted for 71% of the isolates [14].

*S. pneumoniae* expressing the same non-PCV7 serotypes and similar clonal types also expanded in countries other than Portugal where the PCV7 vaccine was introduced and these clones were shown to be able to cause human colonization as well as disease [14-19].

The present study focused on three drug resistant non-PCV7 clones isolated from the nasopharynx of healthy vaccinated children in Portugal [14]. Using several murine models, we examined the behavior of the 6A, 15A and 19A non-PCV7 clones in colonization and disease. Our data show that these three clones were highly competent in host colonization and were able to asymptomatically invade several tissues but remained poorly virulent in the mouse septicemia model and completely avirulent in the pneumonia model.

## Materials and Methods

### Ethics statement

All animal experiments were conducted with the approval of the Rockefeller University Institutional Animal Care and Use Committee (Permit Number: 09073).

### Bacterial strains

The three drug resistant pneumococcal strains, expressing capsular types 6A, 15A and 19A, were recovered from the nasopharynx of healthy PCV7-immunized children in Portugal [14]. The strains were characterized for serotype, multi-locus sequence type (MLST) [20], susceptibility to antibiotics (penicillin, ceftriaxone, oxacillin, chloramphenicol, erythromycin, clindamycin, tetracycline, trimethoprim-sulfamethoxazole and levofloxacin) and pulsed-field gel electrophoresis (PFGE) type as described previously [14]. PFGE-based clonal types were defined as isolates with ≥80% relatedness using the Bionumerics software (Applied-Maths, Sint-Martens-Latem, Belgium). The three pneumococcal strains chosen for detailed study were selected on the basis of three criteria which included: i) serotype; ii) PFGE clonal type and iii) resistance to penicillin (Table 1 and Figure S1 in File S1).

**Table 1:** Major drug resistant non-PCV7 clones.

				**Representative strains**		
**Serotype**	**PFGE clonal type**		**Penicillin MIC associated**	**PFGE**	**Antibiotype**	**ST**
6A	6A-I		P(I) clone, MIC: 0.19 µg/mL	**6A-I**	**P(I**)** (MIC: 0.19 µg/mL**)**, Tet**	**2191**
	6A - II		P(S) clone, MIC range: 0.023-0.064 µg/mL			
15A	15A-I		P(I) clone, MIC range: 0.125-0.19 µg/mL	**15A-I**	**P(I**)** (MIC: 0.19 µg/mL**)**, Tet, Da, E**	**63**
	15A-II		P(S) clone, MIC: 0.016 µg/mL			
19A	19A-I		P(I) clone, MIC range: 0.5-0.75 µg/mL	**19A-I**	**P(I**)** (MIC: 0.75 µg/mL**)**, SXT, Tet, Da, E**	**276**
	19A-II		P(I) clone, MIC: 0.19 µg/mL			
	19A-III		P(I) clone, MIC: 0.094 µg/mL			
	19A-IV		P(S) clone, MIC range: 0.016-0.023 µg/mL			

P(S) – Penicillin Susceptible (P<0.094 µg/ml); P(I) – Penicillin Intermediate Resistance (0.094 µg/ml≤P<1.5 µg/ml) ; E – Erythromycin resistance (E≤15 µg/ml); Da – Clindamycin resistance (Da ≤15 µg/ml); Tet – Tetracycline resistance (Te ≤18 µg/ml); SXT - Trimethoprim-sulfamethoxazole resistance (SXT ≤15 µg/ml).

MIC – Minimal Inhibitory Concentration.

Transformants expressing the type 3 capsule were generated using 6A and 19A as the parental strains. The transforming DNA was from strain SV35T3, which was generated by transforming the SV35-T23 strain with a 22kB region from SV36T3 that contained genes for the type 3 capsule as well as a spectinomycin cassette to create a selectable marker [21]. Each of the two type 3 transformants was backcrossed three times to avoid recombination with unlinked noncapsular genes from the SV35T3 donor producing essentially isogenic strains. The D39 serotype 2 isolate was used as an arbitrary control strain in some animal experiments. Bacteria were grown without aeration at 37°C either in C+Y broth [22], Todd-Hewitt broth supplemented with 0.5% yeast extract (THY) or in Tryptic Soy agar (TSA) containing 5% defibrinated sheep blood supplemented with gentamicin (5 µg/mL) in a 5% CO_2_ atmosphere. Bacterial inocula were prepared by growing pneumococci in C+Y broth to an optical density of 0.8 (OD_590_). Cultures were then centrifuged and the pellets resuspended in a sterile saline solution to obtain the desired bacterial concentration.

### Capsular switching methods

Competent cells were prepared by growing bacteria in THY broth to OD_590_=0.07-0.08. To carry out transformation, competent cells were diluted 1:20 in competence medium (TSB [pH 8.0], 0.16% bovine serum albumin, 0.01% CaCl_2_) containing whole genome transforming DNA (1,000 ng/mL) and the competence stimulating peptide (CSP, ~500 ng). The transforming DNA was purified from strain SV35T3 [21], and CSP1 and CSP2 were used to transform pneumococcal strains 6A and 19A, respectively. The transformation reaction (1 mL) was held 4 h at 37°C in a 1.5-mL Eppendorf tube and then challenged in TSA plates supplemented with 5% sheep blood and spectinomycin (125 µg/mL). Plates were incubated overnight at 37°C with 5% CO_2_. Transformants were confirmed by observation of the typical type 3 mucoid colony morphology, serotyping with specific sera [23] and detection of capsular type 3 by PCR amplification [24].

### Mouse models

Groups of 9-week-old female CD1 outbred mice obtained from the Charles River Laboratories (Wilmington, MA) were used in the colonization, pneumonia and septicemia models. When required, mice were anaesthetized by injecting 75 to 100-µL of a xylazine and ketamine mixture into the peritoneal cavity (0.2 ml Xylazine at 100 mg/mL and 1 ml of Ketamine at 100 mg/mL was mixed with 4.8 ml PBS buffer pH 7.4). The protocol for the animal experiments was approved by the Institutional Review Board of The Rockefeller University (Permit Number: 09073). Colony-forming units (CFU) inoculated in the mice were confirmed by counting of serial dilutions on TSA containing 5% blood supplemented with gentamicin. Mice were given food and water *ad libitum*, and monitored for survival on a daily basis during the experimental period. For sampling procedures mice were humanely euthanized by CO_2_ asphyxiation.

### Colonization model

Each anesthetized mouse was inoculated intranasally with 10 µL of inoculum containing 10^8^ CFU using a 20-µL micropipette. At defined time points after the challenge (2, 7, 14, and 21 days) groups of mice were euthanized and bacterial numbers assessed in the nasopharyngeal and middle ear mucosa, olfactory bulbs, brain, lungs and blood. The nasopharyngeal wash was performed by cannulation of the mouse trachea and posterior lavage with 500-µL of saline solution, which was collected through the nose. To sample the middle ear mucosa, we adapted the procedure used by Lai and colleagues [25]. In brief, we injected into each middle ear cavity 10 µL of sterile saline solution using a 10-µL micropipette. The saline solution was withdrawn and reintroduced three times (final volume recovered, ~13-µL per 2 ears). Blood collection (300 µL) was achieved by heart puncture. Using a tissue homogenizer (PYREX® Potter-Elvehjem), the lungs and brain or the olfactory bulbs were homogenized respectively in 1000 or 500 µL of sterile saline solution. Resulting solutions from all samples were diluted and plated for CFU counting. Mouse perfusion was performed before the lungs, olfactory bulbs and brain were harvested to avoid possible CFU contamination from the blood.

### Pneumonia model

Each anesthetized mouse was inoculated intranasally with 50 µL of inoculum containing 10^5^ or 10^7^ CFU using a 100-µL micropipette and survival rates were followed for 7 days.

### Septicemia model

Each mouse was injected intraperitoneally with 500-µL of inoculum containing 10^5^ CFU and survival rates were followed for 7 days. At defined time points after the intraperitoneal injection (0.5, 3, 6, 12 and 24 h), 1-3 mice were euthanized and bacterial numbers assessed in the blood.

### Statistics

Significant differences in CFU numbers were analyzed using the 2-tailed Mann-Whitney U test. Survival curves were analyzed using the log-rank (Mantel-Cox) test using Prism software from GraphPad Software Inc. In all analyses, a maximum error type I of 0.05 was considered for recognition of a significant difference.

### Whole genome sequencing and assembly of the non-PCV7 strains

The genomes for strains 6A, 15A and 19A were sequenced using the 454 Life Science’s Titanium platform. The depth of coverage was as follows: type 6A: 36.3X coverage; type 15A: 33.9x coverage; type 19A: 37,5X coverage.

Fragment libraries and sequencing were performed following the manufactures’ guidelines outlines in *GS FLX Titanium, General Library Preparation Method Manual* (October 2008 Roche Molecular Systems, Nutley, NJ) and *GS FLX Titanium emPCR and Sequencing Protocols* (October 2008). The raw sequence reads were assembled into contigs using the Roche/454 Life Sciences *GS de novo* Newbler Assembler v2.3. The final assemblies for 15A, 6A and 19A contain 70, 87 and 49 contigs, respectively. The whole genome sequence for 15A, 6A, and 19A are currently being processed in GenBank and accession numbers will be available shortly.

### Phylogenetic analysis

The three newly sequenced non-PCV7 strains were compared to a set of 56 fully sequenced *S. pneumoniae* strains listed in Table S1 in File S1.

### Whole genome sequence alignment and phylogenetic tree

The whole genome sequence of the 59 strains was aligned using MAUVE under default parameters [26], and the core regions were extracted from the xmfa alignment file. The 1.5Mb core region of each of the 59 genomes was aligned using Mafft [27]. This alignment was used to compute a Maximum Likelihood phylogenetic tree using the GTRGAMMA model in RAxML [28], and branch support was computed with 1000 bootstrap replicates. The tree was visualized with FigTree software [29].

## Results

### PCV7 selects strains with non-PCV7 capsules and specific drug resistance traits

The PCV7 vaccine became available in Portugal in July 2001. In a three-year (2001-2003) surveillance study, a group of 238 PCV7-vaccinated children attending day-care in the Lisbon area was investigated regarding the impact of the PCV7 vaccine on colonization [14].

The PCV7 vaccine did not change the overall pneumococcal carriage rate (68.2% in the vaccines and 67.6% in the control) and importantly did not change the carriage rate of drug resistant strains (37.4% in the vaccines and 39.3% in the control). We focused on the latter finding and concluded that the PCV7 vaccine led to a replacement of drug resistant strains expressing PCV7 serotypes by drug resistant strains exhibiting non-PCV7 capsular types.

Among the non-PCV7 drug resistant strains the most prevalent serotypes were 15A (26 strains), 19A (22 strains) and 6A (6 strains), which together accounted for 71% (54/76) of the isolates [14]. Table 1 and Figure S1 in File S1 summarize the genetic diversity among these three major drug resistant non-PCV7 serotypes. Strains expressing serotypes 6A or 15A belonged to one of two PFGE clonal types, while strains that exhibited the 19A capsule belonged to one of four PFGE lineages. Furthermore, among the strains expressing the 6A serotype, the highest penicillin MIC (0.19 µg/mL) was associated with a single PFGE type named 6A-I. For serotype 15A, the highest penicillin MIC (0.19 µg/mL) was associated with the PFGE clone 15A-I, while for serotype 19A the PFGE clone presenting the highest penicillin MIC (0.75 µg/mL) was 19A-I. We next selected representative strains belonging to these specific PFGE types and characterized the isolates by MLST. The clone expressing the 6A serotype belonged to ST2191, the 15A serotype to ST63 and the 19A capsular type to ST276 (Table 1 and Figure S1 in File S1). These three clonal types became the most frequent drug resistant sequence types colonizing the nasopharynx of PCV7-immunized children in Portugal [14].

### Whole genome sequence analysis of the three drug resistant non-PCV7 clones

**Figure 1:**
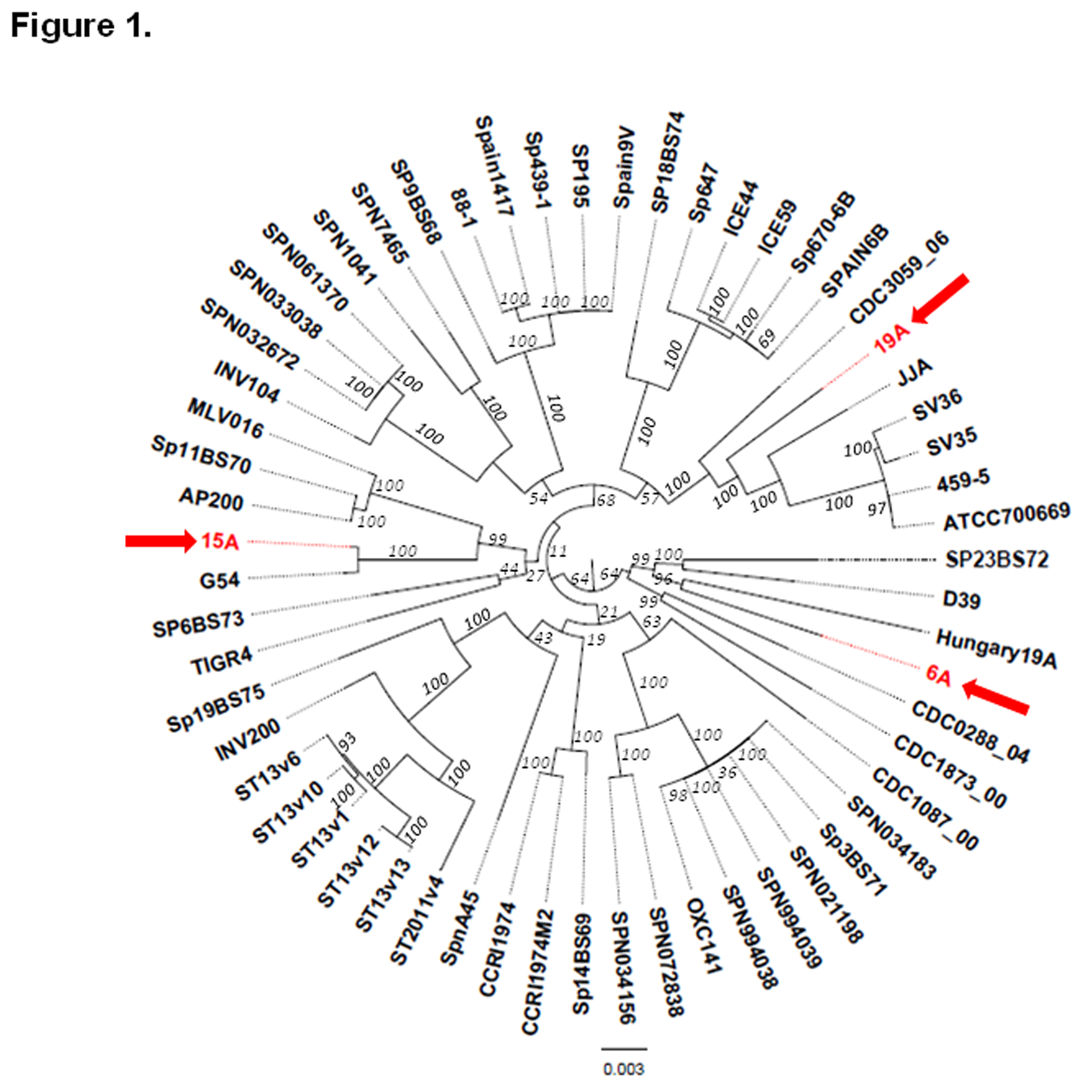
Maximum likelihood phylogenetic tree based on the core genome polymorphisms of 59 *Streptococcus pneumoniae*. Branches are annotated with their bootstrap support (numbers in italics). Three non-PCV7 clones (6A, 15A and 19A) are highlighted in red. Strain 15A is highly similar to G54, strain 6A and 19A are not highly similar to any other strains in this set.

To identify differences among the three non-PCV7 clones and place them in a species-wide perspective, we sequenced the full genomes and performed a phylogenetic analysis of the 6A, 15A and 19A clonal isolates and another 56 *S. pneumoniae* strains that capture much of the species diversity. The whole genome sequences of the 56 pneumococcal strains plus the three non-PCV7 clonal isolates were aligned generating a 1.5Mb region that is shared by all the isolates (referred to as the core WGS). Given that the average pneumococcal genome is 2.1Mb, this shows that ~71% of the genome of each individual strain is core [30]. This alignment-based method does not allow for gaps in any of the sequences, thus we selected the core set to maximize the number of informative positions. The core WGS from each genome was aligned using Mafft, and a Maximum Likelihood phylogenetic tree was computed using RAxML (Figure 1). The relative position of each non-PCV7 clone is supported by high bootstrap values in the inner branches. The 15A lineage is very closely related to G54, a drug resistant 19F clinical isolate, both presenting ST63 [31]. This high degree of similarity in the core region in combination with a difference in capsule type is consistent with one of these clones having evolved by serotype switching. In contrast, the 6A and 19A clones were not highly related to any of the remaining strains included in the currently available whole genome data set (Figure 1). The closest neighbor to the 6A clone is the Hungary-19A clinical isolate (ST268). The 19A clone is in the same branch as strains CDC3059_6 (ST199 clinical isolate), JJA (ST66 clinical isolated from Brazil), and the PMEN1 lineage (ST81). While it is possible that these strains have evolved by serotype switching, this data set did not capture any closely related strains. By MLST, the 15A clone is a representative of Sweden15A-ST63 international clone, the 19A is a SLV of Denmark14-ST230 international clone and for the 6A clone no obvious close relatives have been described but a double locus variant (DLV) has been identified in Greece.

Whole genome sequencing allowed us to check for the presence of major genetic determinants known to play a role in several important processes such as, colonization, disease, competition, antibiotic resistance and natural transformation (Tables 2 and 3) [32-36]. Overall, in the three non-PCV7 clones the same set of genetic determinants was observed with the sole differences noted concerning capsular type and antibiotic resistance pattern. The rlrA pathogenicity island encoding pilus-like structures was missing from each of the three non-PCV7 clones.

**Table 2:** *Streptococcus pneumoniae* genetic determinants involved in colonization, competition, disease, antibiotic resistance and natural transformation.

		**Non-PCV7 clones**		
**Pneumococcal genetic determinants**	**Main role**	**6A**	**15A**	**19A**
		**ST2191**	**ST63**	**ST276**
**Colonization**				
Capsule	Prevents entrapment in the nasal mucus, thereby allowing access to epithelial surfaces. Also inhibits effective opsonophagocytosis.	P (6A)	P (15A)	P (19A)
Choline binding protein (*pspC*)	Binds to human secretory component on a polymeric Ig receptor during the first stage of translocation across the epithelium.	P	P	P
Choline binding protein (*pcpA*)	Role in pneumococcal adhesion and biofilm formation.	P	P	P
*nanA*, *bgaA* and *strH*	Act sequentially to cleave terminal sugars from human glycoconjugates, which might reveal receptors for adherence.	P	P	P
*hyl*	Breaks down hyaluronan-containing extracellular matrix components.	P	P	P
*pavA*	Binds to fibronectin.	P	P	P
*eno*	Binds to plasminogen.	P	P	P
*rlrA* pathogenicity islet	The *rlrA* islet encodes pili-like structures contributing to adherence.	M	M	M
**Competition in upper airway**				
Bacteriocin Locus (*blpC* and *βlpH*)	Small antimicrobial peptide that targets members of the same species.	P	P	P
**Disease**				
*ply*	Cytolytic toxin that also activates complement. An important determinant of virulence in *in vivo* models of disease. Wide range of effects on host immune components at sub-lytic concentrations.	P	P	P
*pspA*	Prevents binding of C3 onto pneumococcal surface. Also binds lactoferrin.	P	P	P*
*lytA*	Digests the cell wall, which results in the release of *ply*.	P	P	P
*psaA*	Component of the ABC transport system, which is involved in resistance to oxidative stress.	P	P	P
*piaA* and *piuA*	Component of the ABC transport system.	P	P	P
*nanA* and *nanB*	Aid colonization by revealing receptors for adherence, modifying the surfaces of competing bacteria that are within the same niche and/or modifying the function of host clearance glycoproteins.	P	P	P
*igA*	Cleaves human IgA1.	P	P	P
*srtA*	Responsible for anchoring most LXPTG-containing proteins.	P	P	P

P=present; M=missing. * small coverage over multiple contigs. Likely to be present in the contig gaps [32-36].

**Table 3:** *Streptococcus pneumoniae* genetic determinants involved in colonization, competition, disease, antibiotic resistance and natural transformation (continued).

		**Non-PCV7 clones**		
**Pneumococcal genetic determinants**	**Main role**	**6A**	**15A**	**19A**
		**ST2191**	**ST63**	**ST276**
**Nature of the drug resistance genes**				
Tetracycline (*tetM*)	Resistance to tetracycline.	P	P	P
Clindamycin (*ermB*)	Resistance to clindamycin.	M	P	P
Erythromycin (*ermB*)	Resistance to erythromycin.	M	P^#^	P
Penicillin-binding proteins (*pbp2X,pbp1A, pbp1B, pbp2A, pbp2B*)	Resistance to penicillin.	P	P	P
**Natural transformation**				
Competence operon (*comABCDE*)	Development of a competence state enabling DNA uptake for genetic transformation.	P	P	P

P=present; M=missing. ^#^ over multiple contigs with gaps [32-36].

### The non-PCV7 clones are highly competent in colonizing and invading adjacent tissues asymptomatically

A murine colonization model was used to assess the capacity of the three major non-PCV7 clones to colonize the mouse nasal mucosa and invade adjacent tissues. To this end, mice were intranasally challenged with a 10-µL inoculum of each of the clones and were followed for a period of three weeks.

The 6A (ST2191) clone was observed in the nasopharynx at all time points over the 21-day experimental period, being also very often isolated from the nasopharyngeal adjacent tissues (lungs, olfactory bulbs, brain and middle ear), and was never present in the blood (Table 4). Out of 24 mice sacrificed, 79% showed the 6A clone colonizing the nasopharynx, while 71% presented it in the olfactory bulbs, 42% in the brain, 21% in the middle ear and 17% in the lungs (Table 4).

**Table 4:** Colonization and infection with the 6A clone.

	Colonization and infection^1^						
Time (Days)	Mice	Nasopharynx	Olfactory bulbs	Brain	Lungs	Middle ear	Blood
2	5	100%	100%	60%	60%	60%	0%
7	6	100%	100%	83%	0%	17%	0%
14	6	67%	50%	0%	0%	17%	0%
21	7	57%	43%	29%	14%	0%	0%
Total	24	79%	71%	42%	17%	21%	0%
	Total CFU recovered^2^						
Time (Days)	Mice	Nasopharynx	Olfactory bulbs	Brain	Lungs	Middle ear	Blood
2	5	7.7E+04	1.8E+03	7.8E+01	5.8E+01	3.4E+01	0.0E+00
7	6	2.7E+04	4.3E+02	4.8E+02	0.0E+00	1.8E+00	0.0E+00
14	6	8.8E+02	1.2E+01	0.0E+00	0.0E+00	1.2E+00	0.0E+00
21	7	1.3E+03	5.0E+00	7.9E+00	3.7E+00	0.0E+00	0.0E+00
Total	24	2.7E+04	5.6E+02	1.4E+02	1.6E+01	9.4E+00	0.0E+00

At day 2, 7, 14, and 21 after intranasal inoculation (10µL) of the mice, several tissues (nasopharynx, olfactory bulbs, brain, lungs, middle ear and blood) were sampled to assess for the presence of live pneumococci.

^1^ Percentage of mice presenting pneumococci in the different tissues.

^2^ Average number of CFUs per mouse in the different tissues.

The 15A (ST63) clone was found in the nasopharynx, olfactory bulbs, brain, lungs and middle ear at all time points but was absent from the blood (Table 5). Out of 24 mice sacrificed, 96% had the 15A clone colonizing the nasopharynx, 75% presented it in the olfactory bulbs, 67% in the lungs, 54% in the brain and 46% in the middle ear (Table 5).

**Table 5:** Colonization and infection with the 15A clone.

	Colonization and infection^1^						
Time (Days)	Mice	Nasopharynx	Olfactory bulbs	Brain	Lungs	Middle ear	Blood
2	6	100%	100%	100%	83%	83%	0%
7	6	100%	83%	67%	83%	50%	0%
14	6	100%	67%	33%	83%	17%	0%
21	6	83%	50%	17%	17%	33%	0%
Total	24	96%	75%	54%	67%	46%	0%
	Total CFU recovered^2^						
Time (Days)	Mice	Nasopharynx	Olfactory bulbs	Brain	Lungs	Middle ear	Blood
2	6	9.2E+04	1.9E+03	4.4E+02	1.2E+04	1.3E+01	0.0E+00
7	6	6.9E+04	5.1E+02	1.4E+02	4.8E+04	3.3E+00	0.0E+00
14	6	1.3E+04	6.6E+01	1.9E+01	8.2E+03	1.8E+00	0.0E+00
21	6	1.4E+04	4.2E+01	1.8E+01	3.4E+01	1.8E+00	0.0E+00
Total	24	4.7E+04	6.2E+02	1.5E+02	1.7E+04	4.9E+00	0.0E+00

At day 2, 7, 14, and 21 after intranasal inoculation (10µL) of the mice, several tissues (nasopharynx, olfactory bulbs, brain, lungs, middle ear and blood) were sampled to assess for the presence of live pneumococci.

^1^ Percentage of mice presenting pneumococci in the different tissues.

^2^ Average number of CFUs per mouse in the different tissues.

The 19A (ST276) clone was detected in the nasopharynx at all time points over the 21-day study. Concomitantly, the 19A clone was frequently isolated from the nasopharyngeal adjacent tissues (olfactory bulbs, brain, lungs and middle ear), while in the blood it was never found (Table 6). Out of 23 mice sacrificed, 78% showed the 19A clone colonizing the nasopharynx, 70% presented it in the olfactory bulbs, 48% in the middle ear and 35% in the brain and lungs (Table 6).

**Table 6:** Colonization and infection with the 19A clone.

	Colonization and infection^1^						
Time (Days)	Mice	Nasopharynx	Olfactory bulbs	Brain	Lungs	Middle ear	Blood
2	5	100%	100%	100%	80%	100%	0%
7	6	100%	100%	50%	50%	67%	0%
14	6	83%	67%	0%	17%	33%	0%
21	6	33%	17%	0%	0%	0%	0%
Total	23	78%	70%	35%	35%	48%	0%
	Total CFU recovered^2^						
Time (Days)	Mice	Nasopharynx	Olfactory bulbs	Brain	Lungs	Middle ear	Blood
2	5	7.9E+04	3.2E+03	1.2E+03	1.6E+02	1.5E+02	0.0E+00
7	6	4.9E+04	4.3E+02	1.2E+02	5.1E+01	1.4E+01	0.0E+00
14	6	3.6E+03	7.5E+00	0.0E+00	1.8E+01	1.8E+00	0.0E+00
21	6	1.0E+03	3.3E+00	0.0E+00	0.0E+00	0.0E+00	0.0E+00
Total	23	3.3E+04	9.0E+02	3.4E+02	5.7E+01	4.2E+01	0.0E+00

At day 2, 7, 14, and 21 after intranasal inoculation (10µL) of the mice, several tissues (nasopharynx, olfactory bulbs, brain, lungs, middle ear and blood) were sampled to assess for the presence of live pneumococci.

^1^ Percentage of mice presenting pneumococci in the different tissues.

^2^ Average number of CFUs per mouse in the different tissues.

The capsular type 2 strain D39 was used as an arbitrary control in all these experiments. The D39 strain was found in the nasopharynx and the adjacent tissues at low CFU numbers, with the exception of the lungs, and was never detected in the blood (Table 7). Out of 10 mice sacrificed, 70% presented the D39 strain in the lungs, 50% presented it in the nasopharynx, 30% in the brain and 10% in the middle ear or the olfactory bulbs (Table 7).

**Table 7:** Colonization and infection with the control D39 strain.

	Colonization and infection^1^						
Time (Days)	Mice	Nasopharynx	Olfactory bulbs	Brain	Lungs	Middle ear	Blood
2	3	100%	33%	33%	67%	33%	0%
7	2	50%	0%	50%	100%	0%	0%
14	3	33%	0%	33%	100%	0%	0%
21	2	0%	0%	0%	0%	0%	0%
Total	10	50%	10%	30%	70%	10%	0%
	Total CFU recovered^2^						
Time (Days)	Mice	Nasopharynx	Olfactory bulbs	Brain	Lungs	Middle ear	Blood
2	3	4.7E+03	1.1E+01	4.0E+00	3.3E+02	8.3E+00	0.0E+00
7	2	6.5E+02	0.0E+00	5.5E+00	1.5E+02	0.0E+00	0.0E+00
14	3	4.0E+00	0.0E+00	4.0E+00	9.3E+03	0.0E+00	0.0E+00
21	2	0.0E+00	0.0E+00	0.0E+00	0.0E+00	0.0E+00	0.0E+00
Total	10	1.3E+03	2.7E+00	3.4E+00	2.4E+03	2.1E+00	0.0E+00

At day 2, 7, 14, and 21 after intranasal inoculation (10µL) of the mice, several tissues (nasopharynx, olfactory bulbs, brain, lungs, middle ear and blood) were sampled to assess for the presence of live pneumococci.

^1^ Percentage of mice presenting pneumococci in the different tissues.

^2^ Average number of CFUs per mouse in the different tissues.

For all clones and in virtually all cases, when mice presented pneumococci in the brain, higher or similar CFU numbers were also found in the nasopharynx and olfactory bulbs of the experimental animals (data not shown). Although all the non-PCV7 clonal lineages tested in this colonization model were able to invade and persist in tissues adjacent to the nasopharynx, no mice presented any signs of disease (e.g. piloerection, eyelid closure or hunched posture) during the 21 days of the experiment (Tables 4-6).

**Figure 2:**
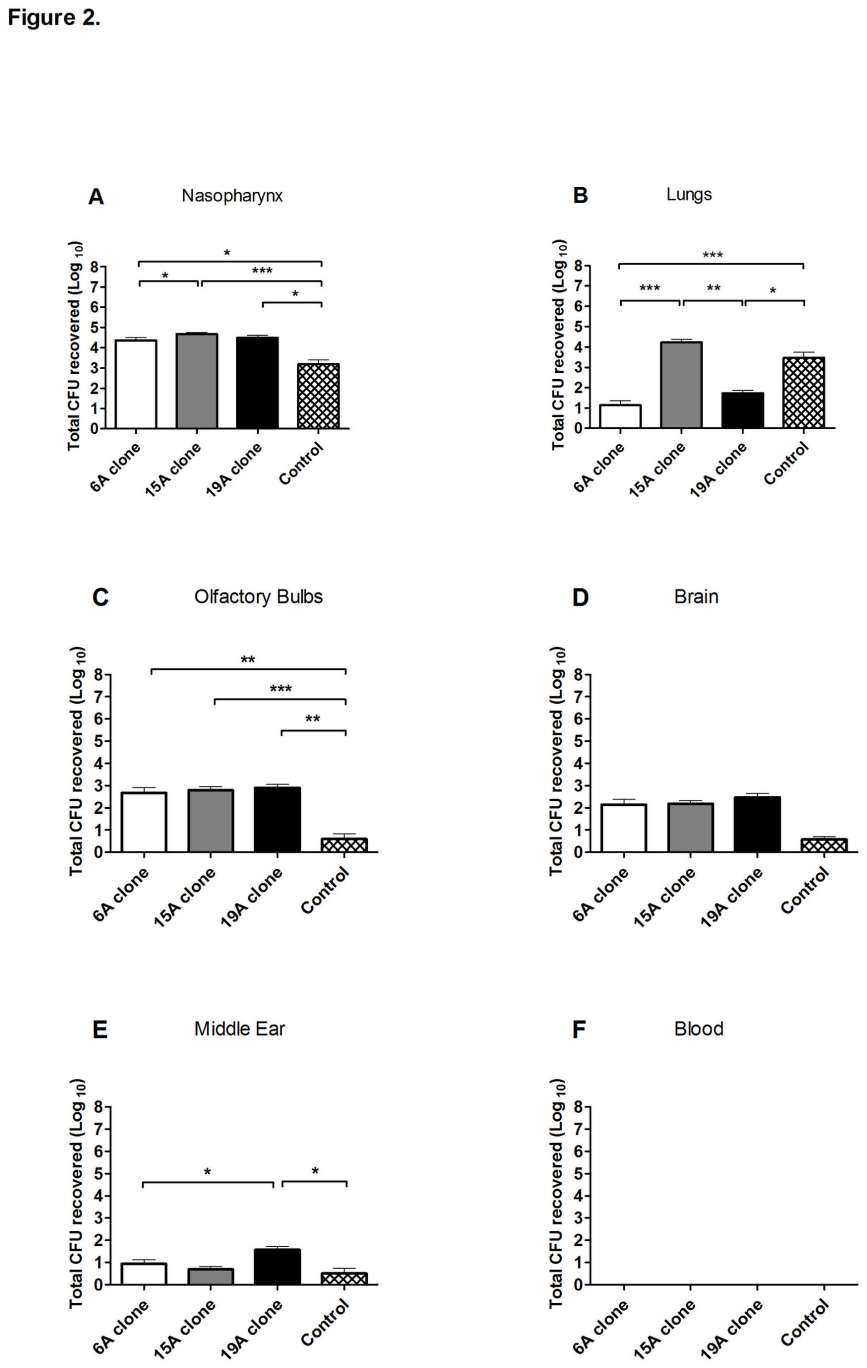
Murine colonization model: persistence of three antibiotic resistant *S. pneumoniae* clones in mouse tissues. Mice were intranasally inoculated with 10^8^ CFU of the non-PCV7 clones (6A, 15A or 19A) or the control strain (D39). At days 2, 7, 14, and 21 after inoculation, CFU counting was performed in the nasopharynx, olfactory bulbs, brain, lungs, and middle ear. Each bar represents the mean of the total CFU recovered (Log_10_) throughout the 21-day experiment for each strain in 10-24 mice ± SEM. (A) nasopharynx, (B) lungs, (C) olfactory bulbs, (D) brain, (E) middle ear and (F) blood. The 0 value on the Y-axis represents the absence of detectable CFUs. The asterisks indicates significant differences, * p<0.05, ** p<0.01 and *** p<0.001 according to 2-tailed Mann-Whitney U test.

In general, the three non-PCV7 clones exhibited similar potential to asymptomatically colonize, invade and persist within the nasopharynx’s adjacent tissues (Figure 2). Along the 21-day experiment the non-PCV7 clones presented a mean of ~10^4^ CFUs recovered from the nasopharynx, while the control D39 strain presented significantly lower CFU counts (p<0.05). In the lungs, the 15A and D39 strains presented significantly higher CFU counts than the 6A and 19A strains (p<0.05). Regarding the olfactory bulbs, the three non-PCV7 clones presented significantly higher CFU numbers than D39 strain, while in the brain no significant differences were found. Lastly, in the middle ear, 19A presented the highest mean CFU numbers (p<0.05) (Figure 2).

Taken together, the above results indicate that the three non-PCV7 clones specialized in colonizing the host nasopharyngeal surface accompanied by asymptomatic invasion and persistence in neighbouring tissues.

### The non-PCV7 clones show none or poor virulence, in the pneumonia and septicemia models respectively

To investigate the potential of the three non-PCV7 clonal types to cause lung infection, we used a murine pneumonia model. Mice were challenged intranasally with a 50-µL inoculum of either the non-PCV7 clones or the control D39 strain and survival was assessed over a 7-day period. Using 10^5^ or 10^7^ CFU inocula, the non-PCV7 clones appeared to be harmless, causing no death or any significant sign of disease (e.g. piloerection, eyelid closure or hunched posture) in the mice challenged. In contrast, using the highly virulent D39 strain in the same range of CFUs caused lethality in all mice (data not shown).

**Figure 3:**
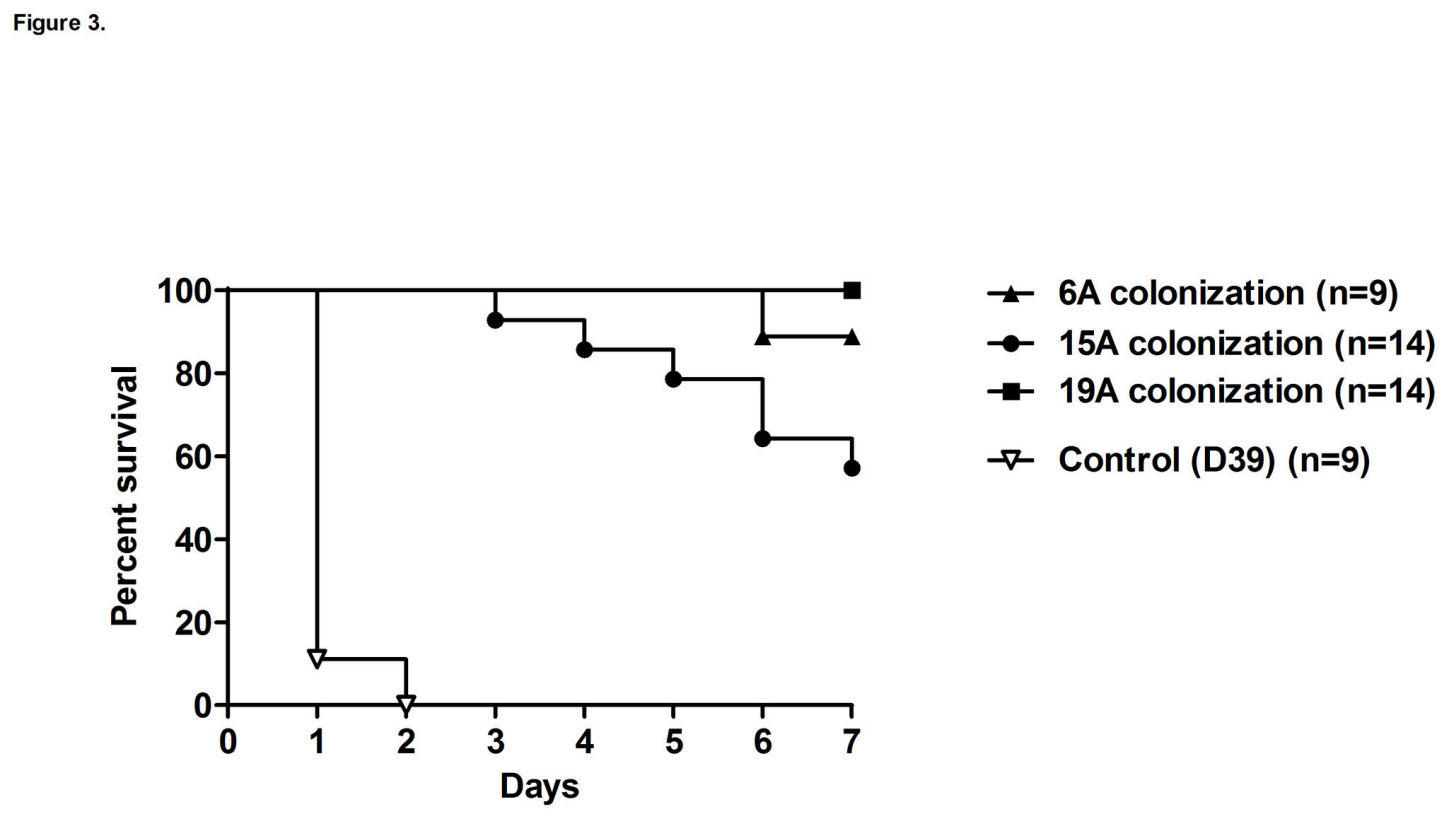
Intraperitoneal murine septicemia model: survival curves. Mice were intraperitoneally challenged with 10^5^ CFU of the non-PCV7 clones, 6A, 15A or 19A and the control strain (D39). Survival was followed to assess the virulence potential of the strains. Survival curves for the non-PCV7 clones were significantly different from the D39 control strain (Mantel-Cox test, p<0.05).

**Figure 4:**
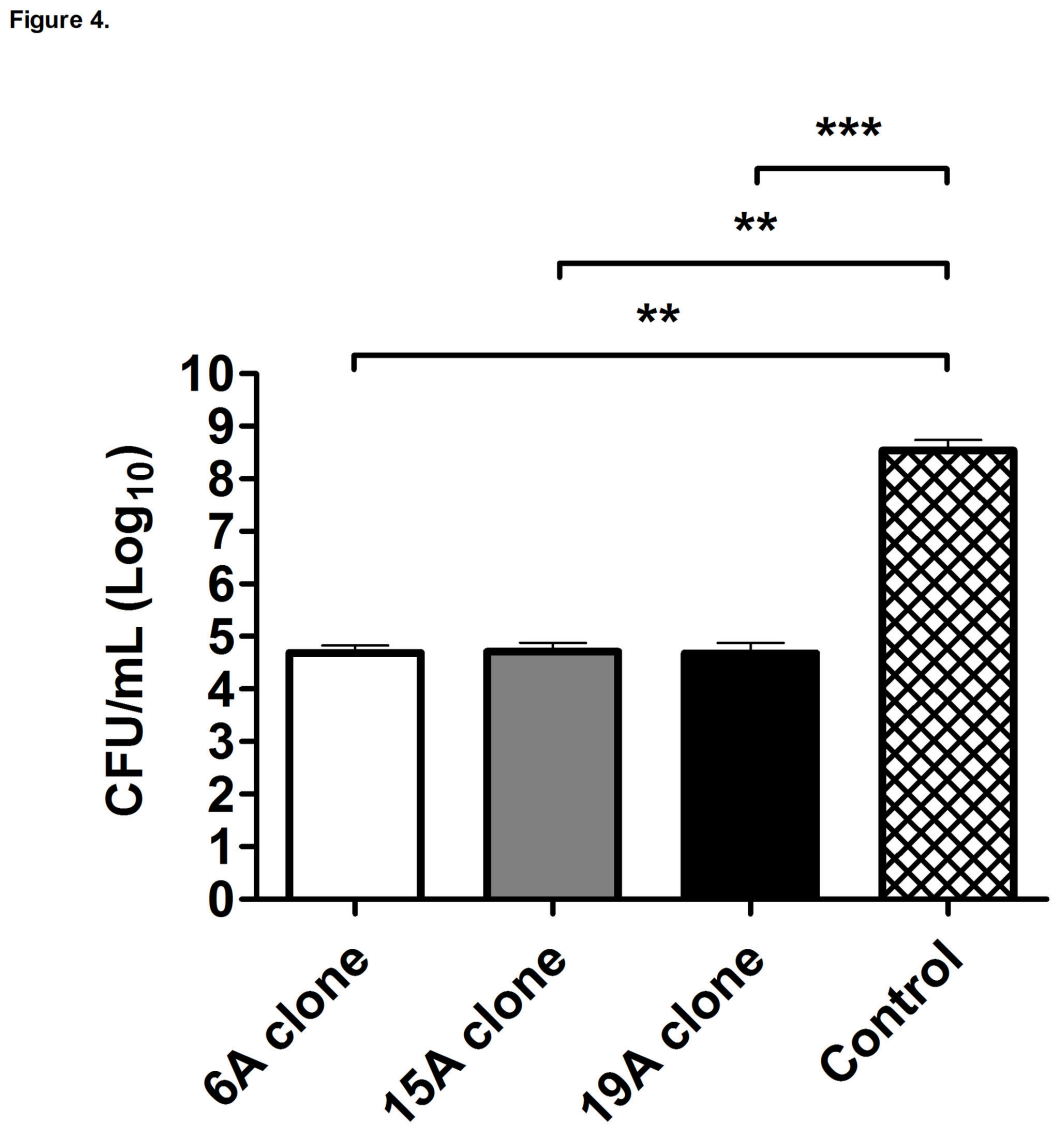
Intraperitoneal murine septicemia model: strain growth. Mice were intraperitoneally challenged with 10^5^ CFU of the non-PCV7 clones (6A, 15A or 19A) or the control strain (D39). At 0.5, 3, 6, 12 and 24 hours after intraperitoneal injection, CFU counting was performed in the blood. Each bar represents the log_10_ of the mean CFU/mL throughout the 24-h experiment for each strain in 13-14 mice ± SEM. The 0 value on the Y-axis represents the absence of detectable CFUs. The asterisks indicates significant differences, ** p<0.01 and *** p<0.001 according to 2-tailed Mann-Whitney U test.

We used an intraperitoneal model to evaluate the potential of non-PCV7 clones to cause septicemia. Mice were challenged with the non-PCV7 clones or the control D39 strain and survival was assessed over a 7-day period. Using a 10^5^ CFU inoculum the non-PCV7 clones were poorly virulent as compared to strain D39 which killed all mice within 2 days of the challenge (Figure 3). Consistent with the results from this survival experiment, we observed that during the first 24 h after the intraperitoneal challenge the D39 control strain already displayed mean titers of 3.5 x10^8^ CFUs per mL of blood, a significantly higher load than that presented by the three non-PCV7 clones (~10^4^ CFU/mL), which were virtually avirulent in this intraperitoneal septicemia model (Figure 4).

### Contribution of capsular type to colonization and disease

**Figure 5:**
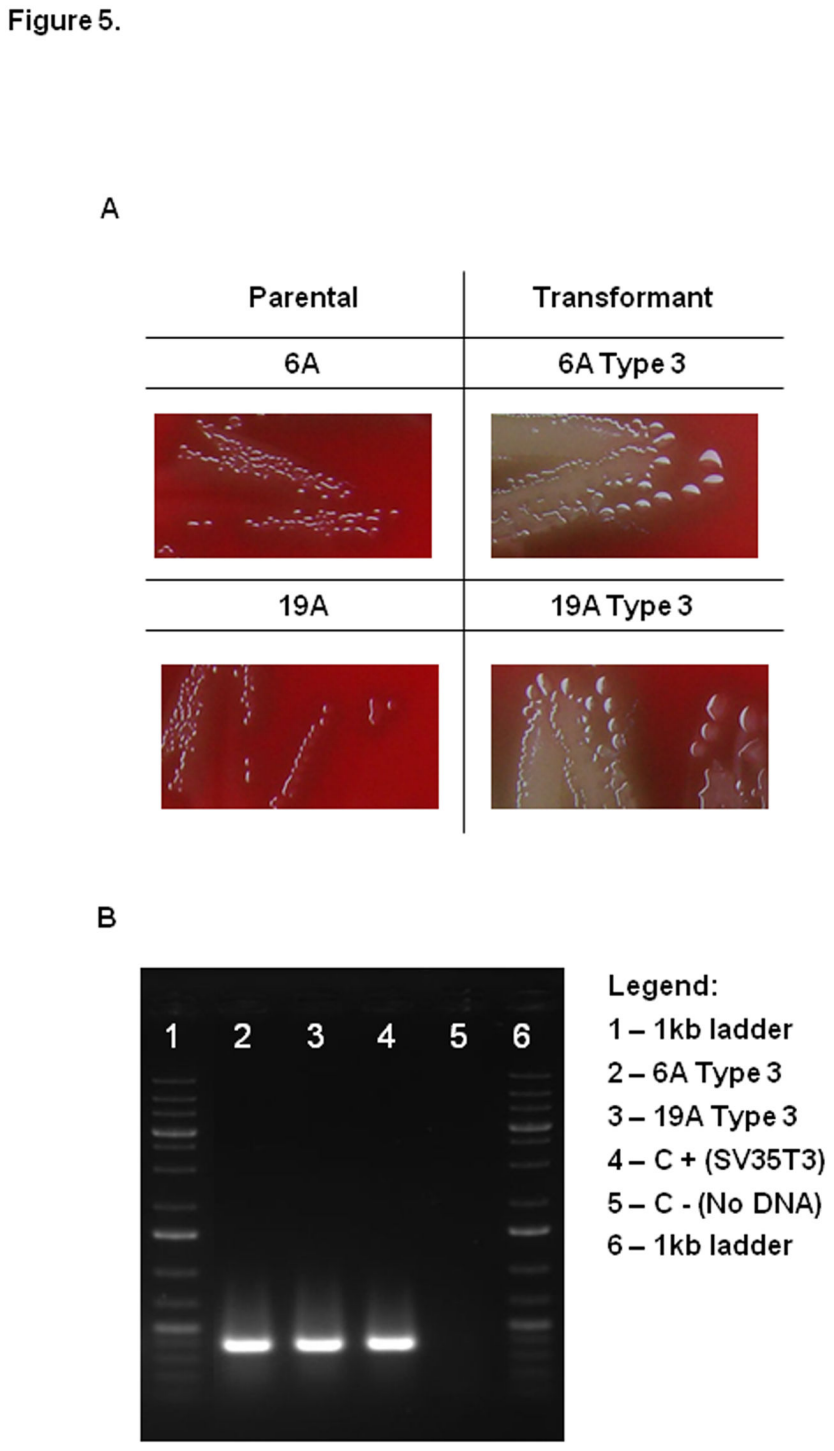
Colony morphology and PCR for detection of capsular type 3. (A) Colony morphology of parental and Type 3 transformant strains. (B) PCR amplification for detection of capsular type 3.

To investigate whether the capsule or the genetic background is the preponderant factor driving the potential of the non-PCV7 clones to colonize or cause disease (septicemia), we generated isogenic strains expressing different capsular types. Two non-PCV7 clones, 6A and 19A, were transformed in the laboratory to express the capsular type 3. Figure 5 shows the morphology of the parental and type 3 transformants (mucoid), as well as PCR amplification confirming the presence of the type 3 operon in the transformants’ genome.

**Figure 6:**
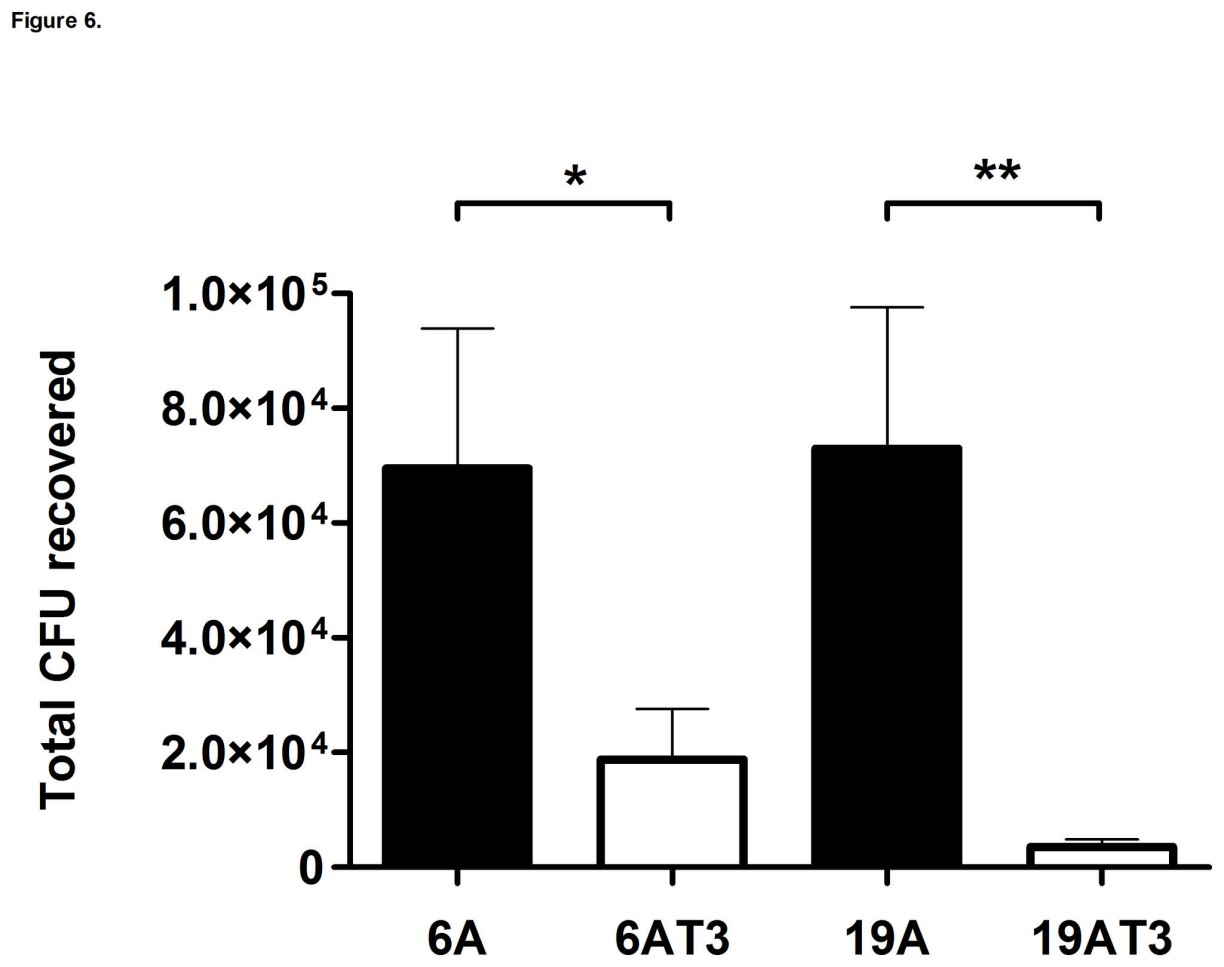
Impact of capsular polysaccharide in colonization. Mice were intranasally challenged with *S. pneumoniae* strains (10^8^ CFU), wild-type (black columns) or the type 3 transformants (white columns) in a 10-µL volume. At day 2, after inoculation, nasal lavage (500 µL) was performed to assess for the presence of live pneumococci. Values represent the mean of the total CFU ± SEM recovered from 5 to 7 mice per strain. The 0 value on the Y-axis represents the absence of detectable CFU. The asterisks indicates significant differences, * p<0.05 and ** p<0.01, according 2-tailed Mann-Whitney U test.

The nasopharynx of mice was colonized with the 6A or 19A clones and with the corresponding type 3 transformants (6A Type 3 and 19A Type 3). At day 2 after the challenge, the CFUs in the nasopharynx of the mice were quantified for comparison (Figure 6). The 6A clone presented on average 7x10^4^ CFU, and the corresponding type 3 a significantly reduced bacterial load (1x10^4^ CFUs) in the nasopharynx (p<0.05). The 19A clonal isolate also colonized each mouse with an average of 7x10^4^ CFU, and the 19A type 3 transformant also showed a significantly lower (p<0.05) average number of CFUs recovered per mouse (3x10^3^) as compared to the wild-type clone (Figure 6).

**Figure 7:**
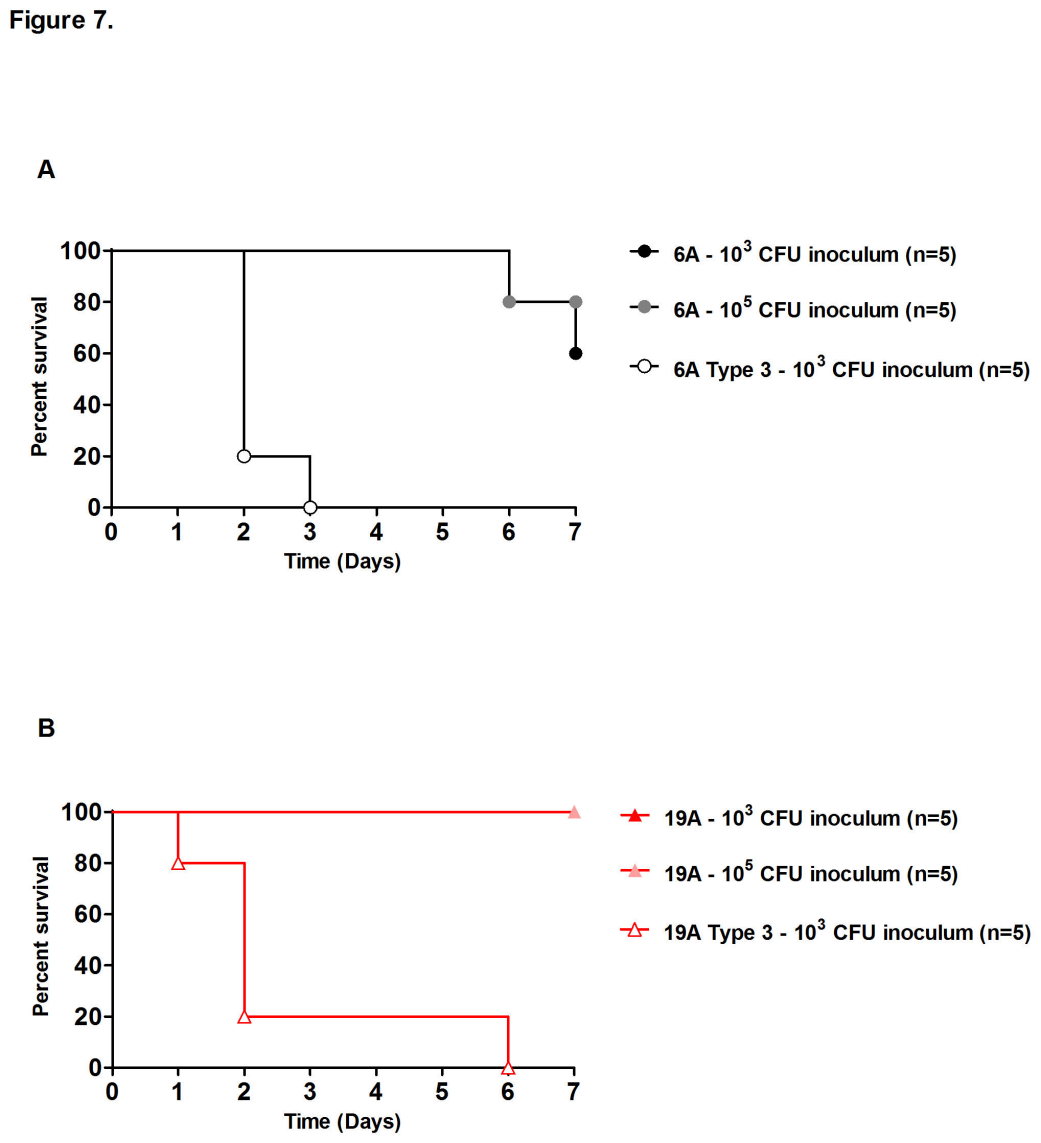
Impact of capsular polysaccharide on survival in a murine model of septicemia. (A) and (B), isogenic strains expressing either capsular types 6A or 19A and the corresponding capsular type 3 transformants were injected into the peritoneal cavity of CD1 mice. Survival was followed to assess the virulence potential of the capsular types. Five mice per serotype/inoculum were tested. Survival curves for parental strains (6A and 19A clones) were significantly different from the corresponding type 3 transformants (Mantel-Cox test, p<0.01).

Next, mice were infected intraperitoneally with the 6A or 19A clones and the corresponding type 3 transformant strains. Following infection, mice survival was assessed over a 7-day period (Figure 7). When the infection inoculum of 6A or 19A was 10^3^ or 10^5^ CFU per mouse, the vast majority of the animals survived the challenge without any signs of disease (e.g. piloerection, eyelid closure or hunched posture). However, when challenged with as few as 10^3^ CFU of the type 3 transformant strains, all animals died before day 7, presenting signs of disease as early as one day after the intraperitoneal challenge (Figure 7). Analysis of the survival curves indicated statistically significant differences (p<0.01) when comparing the parental strain with the corresponding type 3 transformant (Figure 7).

These results indicate that the capsular type can have a major and opposing impact on colonization and disease.

## Discussion

The commercial introduction of the 7-valent anti-pneumococcal conjugate vaccine (PCV7) in Portugal, in 2001, caused an extensive change in the nature of *S. pneumoniae* strains colonizing the nasopharynx of pre-school aged children, which represents the major natural reservoir of this bacterial pathogen [1,37,38]. In a study conducted in Portugal, among PCV7-vaccinated children, drug resistant strains expressing capsular polysaccharides included in the vaccine were replaced by strains that were also drug resistant but produced capsules of different chemical composition [14]. The overwhelming majority (71%) of the “new” drug resistant colonizing strains expressed three serotypes, 6A, 15A or 19A – polysaccharides that are not included in the vaccine. Interestingly, these non-PCV7 serotypes were represented by “novel” PFGE types [14] and molecular typing described in the present communication further identified the major non-PCV7 strains as serotype 6A with sequence type (ST) ST2191, serotype 19A as ST276 and the capsular type 15A as ST63.

As to the possible origin of these non-PCV7 clones we compared them to the genomes of 56 fully sequenced *S. pneumoniae* strains (Figure 1)*.*

The capsular type 15A (ST63) strain was found to only differ from the fully sequenced 19F clinical isolate G54 in the chemical composition of the capsular polysaccharide indicating that this lineage has the capacity to undergo *in vivo* capsular switch. A capsular switch may produce “vaccine escape” recombinants [39] that can avoid the vaccine-induced immune pressure. The importance of such genetic events in the *in vivo* evolution of *S. pneumoniae* has been documented [21]. In Portugal and elsewhere, both 15A and 19F variants of ST63, the Sweden15A-ST63 PMEN international clone [14], had been detected before introduction of PCV7 [14].

As to the 6A (ST2191) and the 19A (ST276) clones, the genomes of the currently available fully sequenced *S. pneumoniae* showed no isolates with comparable sequence identity. However, by MLST, ST276 is a SLV of Denmark14-ST230 clone [14], and this lineage has been in circulation in Portugal since the pre-PCV7 era [14].

The approximate “position” of these three non-PCV7 clones on a phylogenetic tree is shown in Figure 1.

The major purpose of the studies described in this communication was to use the adult mouse models of pneumococcal colonization and disease to evaluate the disease potential of the three major clonal types of non-PCV7 isolates that were selected *in vivo* among vaccine-treated Portuguese children. The main conclusion that emerged from these tests was that the three clones were highly efficient in causing colonization and long-term carriage, but exhibited only poor invasive potential.

Because of the discrepancy between high rate of colonization and poor performance in terms of virulence, it was important to test if the three major non-PCV7 clones were equipped with genetic determinants of known virulence factors. Tables 2 and 3 show that the genetic determinants of each of the major known virulence factors of *S. pneumoniae* were carried by the three non-PCV7 clones. The exception was the rlrA pathogenicity islet or pilus islet-1 (PI-1), mostly prevalent in the pre-PCV7 era among vaccine type strains [40], but absent from the three non-PCV7 clones studied here.

The results of experiments presented in Figure 7 clearly demonstrate that the primary factor determining the degree of virulence in these strains – as measured in the mouse intraperitoneal model – was the chemistry of the capsular polysaccharide. We replaced the 6A and the 19A capsules with the capsular type 3, by genetic transformation in the laboratory and produced type 3 derivatives of the 6A (ST2191) and 19A (ST276) that were able to induce rapid lethal infection in the mouse at low bacterial inocula in the range of 10^3^ CFU per animal – suggesting that the bacterial structure primarily responsible for the lethality of infection was the capsular polysaccharide and the genetic background of the two non-PCV7 clones did not prevent the expression of high degrees of virulence in these laboratory produced capsular type 3 derivatives of the non-PCV7 clones.

Most interestingly, the *in vitro* capsular switch that caused the massive increase in virulence had the opposite effect on the colonizing potential of the same type 3 transformants, which had significantly reduced capacity to colonize the experimental animals (Figure 6). These findings parallel experience with pneumococcal infectious disease in which the most invasive capsular types (capsular type 1, 4, 5, 7F, etc) have rarely been identified in the human colonizing flora [41,42].

Several recent reports [43,44] suggest that the frequency of pneumococcal disease by non-PCV7 isolates is reduced in the post PCV7 era. These findings find a parallel with a low degree of virulence of the three non-PCV7 clones in the mouse models. The serotype 19A (ST276) and 15A (ST 63) clones have been identified as *S. pneumoniae* clonal types most frequently recovered from pneumococcal infections worldwide – in countries that introduced the PCV7 vaccine [15,16,45,46]. For reasons unknown, representatives of the third major colonizing clone with serotype 6A (ST2191) have not been recovered from pneumococcal disease. In contrast, colonization by each of the three major non-PCV7 clonal lineages have been widely reported [14,17,19].

No data are currently available concerning the severity of disease caused by drug resistant non-PCV7 strains. Limited experience with another multi-drug resistant clone – PMEN1 – documented that this clone had the capacity to cause the entire spectrum of pneumococcal diseases and thus appeared to pay “no price” for the drug resistance in terms of a diminished virulence [47].

The major clonal types selected for characterization in this study showed resistance to several antibacterial agents. In a previous study the frequency of resistant strains among colonizing isolates recovered from vaccinated and non-vaccinated children were shown to be comparable. For instance the frequency of penicillin resistance was ~24% among the colonizing pneumococci recovered from vaccinated and non-vaccinated attendees in day-care centers in Portugal [14]. The major clonal types selected for characterization showed resistance to penicillin in the range of 0.19 to 0.75 µg/mL suggesting the presence of mutant *pbp* genes [48]. Whether or not the *murM* determinant previously implied in the mechanism of resistance to penicillin carries mutations remains to be determined in the non-PCV7 strains [49].

The newly emerged non-PCV7 strains carry the same antibiotic resistance mechanisms against tetracycline (*tetM*), clindamycin and erythromycin (*ermB*) that were already identified in the vaccine type strains.

The newly emerged clones also carry the competence operon (Table 3) and acquisition of antibiotic resistance determinants from the majority vaccine type strains through genetic transformation seems to have occurred during the pre-PCV7 era [49]. Clones 6A (ST2191), 19A (ST276) and 15A (ST63) must have pre-existed as minority components of the complex nasopharyngeal flora in the era prior to the introduction of the PCV7 vaccine [9,50] and must have “witnessed” the same selective pressure of antibiotic use that has led to the emergence of drug resistance lineages in the pre-PCV7 era. This notion is consistent with the surprisingly high frequency of drug resistance strains detected among the newly emerged non-PCV7 strains which is similar (about 40%) to the frequency of antibiotic resistance among colonizing strains characterized during the pre-PCV7 era [14,51,52].

We observed in the mouse model of colonization that the three major non-PCV7 clones have the capacity to colonize the nasopharyngeal mucosa for prolonged times and to invade and prevail within neighboring tissue (olfactory bulbs, brain, lungs or the middle ear) without causing lethal infections or any signs of disease in the experimental animals (Figure 2). This latter finding strongly suggests that stable pneumococcal populations can be established within tissues, protecting pneumococci against mucus flow or competition with the microflora present on the nasopharynx’s surface. Pneumococci from within the nasopharynx mucosal tissue [53] or deeper tissues could then constitute a reservoir able to “seed” the nasopharyngeal surface, ensuring recurrent colonization of this mucosa.

Interestingly, and in accordance with previously reported data [54,55], the colonized mice with brain infection did not present bacteria in the blood, clearly showing that pneumococci can enter the brain directly from the nasopharynx bypassing the blood [55].

In the experimental model we used adult outbred mouse. However, pneumococcal disease, particularly otitis media, is a most frequent affliction in children and a more appropriate model to evaluate the disease potential of the three non-PCV7 clones would be to also test their virulence potential in the infant mouse model, ideally in combination with a simultaneous viral infection, which represents the most frequent scenario of human pediatric disease [56]. Experiments evaluating the disease potential of the three non-PCV7 clones in such an infant mouse model are currently in progress.

## Supporting Information

File S1 **Figure S1, PFGE clonal types associated to serotypes 6A, 15A and 19A. Table S1, List of the 59 sequenced *Streptococcus pneumoniae* strains used in the phylogenetic analysis.**
(PDF)
