# Supplementary material for: Virulence Potential and Genome-Wide Characterization of Drug Resistant Streptococcus pneumoniae Clones Selected In Vivo by the 7-Valent Pneumococcal Conjugate Vaccine
Source: PLoS One. 2013 Sep 19;8(9):e74867. doi: 10.1371/journal.pone.0074867 (PMC3777985; doi:10.1371/journal.pone.0074867)
Supplement: File S1 — Figure S1, PFGE clonal types associated to serotypes 6A, 15A and 19A. Table S1, List of the 59 sequenced Streptococcus pneumoniae strains used in the phylogenetic analysis. (PDF) [file pone.0074867.s001.pdf]

SUPPORTING INFORMATION

Figure S1. PFGE clonal types associated to serotypes 6A, 15A and 19A.

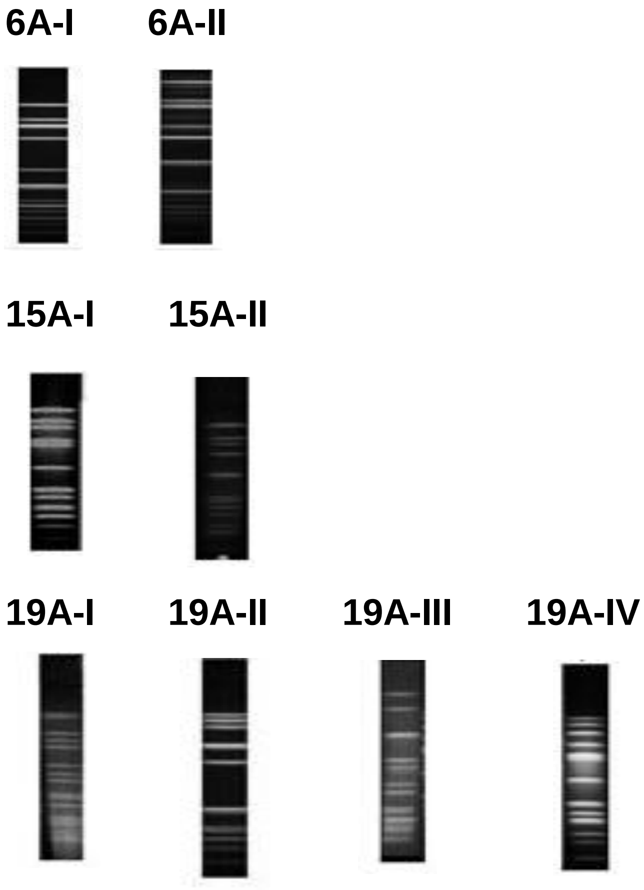

SUPPORTING INFORMATION

Table S1. List of the 59 sequenced *Streptococcus pneumoniae* strains used in the phylogenetic analysis.

Strain	Serotype	MLST	Genome (bp)	Location of isolation	Source	Genbank Accession Number
WL400	6A	2191	2149529	Portugal	carriage	In process.
B605	15A	63	2085829	Portugal	carriage	In process.
WL677	19A	276	2085829	Portugal	carriage	In process.
INV200	14	9	2093318	Oxford, UK	disease	FQ312029
ST13v10	14	13	2063728	Pittsburgh, USA	disease	NZ_ABWA01000000
ST13v13	NT	13	2070802	Pittsburgh, USA	disease	NZ_ABWC01000000
ST13v6	NT	13	2053197	Pittsburgh, USA	disease	NZ_ABWB01000000
ST13v1	14	13	2100368	Pittsburgh, USA	carriage	NZ_ABWQ00000000
ST13v12	NT	13	2065452	Pittsburgh, USA	disease	NZ_ABWU00000000
Sp23BS72	23	37	2103479	Pittsburgh, USA	disease	ABAG00000000
AP200	11A	62	2084139	University of Siena, Italy	disease	CP002121
MLV016	11A	62	2247118	USA, Europe	carriage	ABGH00000000
Sp11BS70	11	62	2060705	Pittsburgh, USA	disease	ABAC00000000
G54	19F	63	2078953	Italy	disease	CP001015
ICE59	6B	63	2212616	Iceland	disease	In process.
Sp647	6B	63	2166935	Spain	disease	In process.
JJA	14	66	2120234	Brazil	disease	CP000919
ICE44	6B	71	2213083	Iceland	disease	In process.
ATCC700669	23F	81	2221315	Spain	carriage	FM211187
4595T23	23	81	2169192	Lisbon, Portugal	carriage	ABXO01000000
SV35T23	23	81	2161785	AIDS clinic of St. Vincent's Medical Center, Richmond, New York, USA	disease	ADNN01000000
SV36T3	3	81	2156372	AIDS clinic of St. Vincent's Medical Center, Richmond, New York, USA	disease	ADNO01000000
670-6B	6B	90	2240045	Spain	disease	CP002176
SPAIN6B	6B	90	2210903	Spain	carriage	In process.
CCRI_1974M2	14	124	2003231	McGill University, Canada	disease	ABZT00000000
CCRI_1974	14	124	2005075	McGill University, Canada	disease	ABZC00000000
Sp14BS69	14	124	2148093	Pittsburgh, USA	disease	ABAD00000000
SP195	9V	156	2198294	Worldwide	disease	ABGE00000000
439-1	9V	156	2065561	Portugal	carriage	In process.
88-1	14	156	2061797	Portugal	carriage	In process.
1417	9V	156	2062281	Portugal	carriage	In process.
SPAIN9V	9V	156	2086592	Spain	disease	In process.
N034156	3	180	2058353	The Netherlands	carriage	FQ312045
N034183	3	180	1993183	The Netherlands	carriage	FQ312043
N994038	3	180	2010908	Glasgow Reference Lab	disease	FQ312041
N994039	3	180	2010104	Glasgow Reference Lab	disease	FQ312044
OXC141	3	180	2036967	Oxford, UK	carriage	FQ312027
Sp3BS71	3	180	2033581	Pittsburgh, USA	disease	AAZZ00000000
SPN021198	3	180	1989367	Glasgow Reference Lab	disease	CACH01000000
SPN072838	3	180	1990038	Bolivia	carriage	CACI01000000
CDC1087	7F	191	2190853	Bra, Den, Fin, Neth, Nor, UK, Uru, USA	disease	ABFT00000000
CDC3059	19A	199	2293277	Iceland, UK, USA, others	disease	ABGG00000000
TIGR4	4	205	2160842	Norway	disease	AE005672
SPN1041	1	217	2166490	Ghana	disease	CACE01000000
CDC0288	12F	220	2051140	USA, UK	disease	ABGF00000000
INV104	1	227	2142122	Oxford, UK	disease	FQ312030
Hungary19A	19A	268	2245615	Hungary	disease	CP000936
N032672	1	306	2144331	Glasgow Reference Lab	disease	FQ312039
N033038	1	306	2164519	Glasgow Reference Lab	disease	FQ312042
SPN061370	1	306	2012346	Glasgow Reference Lab	disease	CACJ01000000
CDC1873	6A	376	2265195	USA	disease	ABFS00000000
Sp6BS73	6	460	2162916	Pittsburgh, USA	disease	ABAA00000000
Sp19BS75	19	485	2136434	Pittsburgh, USA	disease	ABAF00000000
D39	2	595	2046115	USA	disease	CP000410
N7465	1	615	2100988	Type strain, Rockefeller USA, 1948	disease	CACF01000000
Sp9BS68	9	1269	2117908	Pittsburgh, USA	disease	ABAB00000000
ST2011v4	Non Typeable	2011	2086050	Pittsburgh, USA	disease	NZ_ADHN01000000
Sp18BS74	6	New	2105593	Pittsburgh, USA	disease	ABAE00000000
SpnA45	3	New	2041833	Newmarket, Pittsburgh, USA	disease	CACG01000000
